# LncRNA CTD-3252C9.4 modulates pancreatic cancer cell survival and apoptosis through regulating IFI6 transcription

**DOI:** 10.1186/s12935-021-02142-0

**Published:** 2021-08-16

**Authors:** Xin Yin, Jingyan Yang, Jintian Chen, Ruiqi Ni, Yanhao Zhou, Hao Song, Liang Jin, Tingting Tang, Yi Pan

**Affiliations:** 1 State Key Laboratory of Natural Medicines, Jiangsu Key Laboratory of Druggability of Biopharmaceuticals, School of Life Science and Technology, China Pharmaceutical University, 24 Tongjiaxiang Avenue, Nanjing, Jiangsu China; 2 Center for Genetic Medicine, Xuzhou Maternity and Child Health Care Hospital, 46 Heping Road, Xuzhou, Jiangsu China; 3 Cancer Institute, Xuzhou Medical University, 84 West Huaihai Road, Xuzhou, Jiangsu China

**Keywords:** Pancreatic cancer, Long noncoding RNA, Cell apoptosis, IFI6

## Abstract

**Background:**

Pancreatic cancer (PC) is one of the most lethal cancer types with high degree of malignancy and poor prognosis. Recent studies have shown that long non-coding RNAs (lncRNAs) were associated with the initiation and progression of pancreatic cancer. In the current study, we have investigated the expression, biological function and mechanism of a lncRNA CTD-3252C9.4 in pancreatic cancer.

**Methods:**

The expression of CTD-3252C9.4 in pancreatic cancer cells and tissues was measured by qRT-PCR. In vitro and in vivo functional experiments assays were implemented for identifying CTD-3252C9.4 function in pancreatic cancer. Molecular relationships among CTD-3252C9.4, IRF1 and IFI6 were investigated via luciferase reporter assay, pulldown assay and ChIP assays.

**Results:**

CTD-3252C9.4 was found remarkably decreased in pancreatic cancer cells and tissues. Overexpression of CTD-3252C9.4 suppressed migration, invasion and proliferation, yet facilitated apoptosis of pancreatic cancer cells both in vitro and in vivo. Then, IFI6 was identified as a downstream target that could be down-regulated by CTD-3252C9.4 and IFI6 overexpression could counteract the effects of CTD-3252C9.4 upregulation on the survival and apoptosis of pancreatic cancer cells. Furthermore, mechanism experiments revealed that IRF1 was a transcriptional factor of IFI6 that can be blocked by CTD-3252C9.4 to inhibit IFI6 transcription.

**Conclusion:**

Our data indicated that CTD-3252C9.4 could promote pancreatic cancer cell apoptosis and restrain cell growth via binding IRF1 and preventing the transcription of IFI6, which may become a potential therapeutic target for pancreatic cancer.

**Supplementary Information:**

The online version contains supplementary material available at 10.1186/s12935-021-02142-0.

## Background

Pancreatic cancer (PC) is one of the most aggressive tumors associated with exceedingly poor survival and high mortality rate. Currently, pancreatic cancer is the seventh primary cause of global cancer-related deaths. However, it is predicted to become the second leading cause of deaths related with cancers by 2030 [1]. With an only 9% of 5-year relative survival, pancreatic cancer has the lowest survival rate among solid tumors [2]. Therefore, identifying new preventive or curative strategies targeting important molecules which promote pancreatic cancer development based on the novel molecular mechanism is an urge.

Apoptosis and proliferation, as two major mechanisms which regulate pathways to control cell state, are provided for consideration as the targeted therapy strategies for tumor cells [3]. Recent findings have verified that a mitochondrial inner membrane protein, interferon alpha inducible protein 6 (IFI6), is involved in various malignancies, including esophageal squamous cell carcinoma, myeloma, breast and gastric cancers [4, 5]. Despite the knowledge about its biological functions remains limited, IFI6 was characterized as an anti-apoptotic and proliferative factor. Besides, it has been reported that IFI6-induced mitochondrial redox deregulation facilitates breast cancer metastasis [6]. Nevertheless, either the biological functions of IFI6 or the mechanisms for IFI6-mediated effects in pancreatic cancer remain unclear.

Dysregulation of long noncoding RNAs (lncRNAs) has been detected in diverse types of cancers and discovered to be closely related to cancer progression [7]. LncRNAs are a class of non-coding transcripts which are > 200nt in length and are destitute of open reading frames. Through multiple ways, they exert influences on gene expressions, like transcriptional and post-transcriptional as well as epigenetic modifications. Also, the critical regulatory roles played by lncRNAs in the progression of pancreatic cancer is worth mentioning. For example, lncRNA PLACT1 is involved in promoting pancreatic cancer cells proliferation and invasion through activation of NF-κB signaling pathway [8]. Moreover, through regulation of E-cadherin, lncRNA LOC389641 promotes pancreatic cancer development and metastasis [9]. In addition, the mutual feedback of HIF-1α and lncRNA MTA2TR drives hypoxia-induced tumorigenesis of pancreatic cancer by regulating MTA2 expression [10]. The above studies indicate that lncRNAs play broad regulatory roles in pancreatic cancer initiation and progression. Nevertheless, the regulatory mechanisms and functions of lncRNAs in pancreatic cancer need in-depth investigation to provide potential targets for pancreatic cancer treatments.

Our previous high-throughput sequencing (HTS) results revealed that lncRNA CTD-3252C9.4 was down-regulated in Panc-1 spheroid cells that enriched in pancreatic cancer stem cells (PCSCs) which display enhanced tumorigenic potential [11]. According to previous studies, CTD-3252C9.4 (alias LOC284454) was expressed differentially in diverse cancers, such as head and neck, prostate, breast, uterus, ovary and colon cancers [12, 13], however, the biological role of CTD-3252C9.4 in pancreatic cancer and its underlying molecular mechanism remain unclear. Therefore, we first verified the down-regulatory expression profile of CTD-3252C9.4 in pancreatic cancer tissues and cells. Then, we showed that CTD-3252C9.4 suppressed pancreatic cancer cell migration, invasion and proliferation, while promoted cell apoptosis in vitro and in vivo. Mechanistically, we found that IFI6 is a downstream target of CTD-3252C9.4. By binding to the transcriptional factor interferon regulatory factor 1 (IRF1), CTD-3252C9.4 prevents the transcription of IFI6, which ultimately induces pancreatic cancer cell apoptosis and represses cell survival. Our finding provides a novel insight into the pathogenesis of pancreatic cancer and potential targets for pancreatic cancer therapy.

## Materials and methods

### Patients and clinical specimens

We collected 20 pairs of human pancreatic tissues and adjacent (located > 5 cm away from tumor) normal tissue samples were collected from patients with pancreatic ductal adenocarcinoma (PDAC) who went through primary surgical treatment at Zhongda Hospital (Nanjing, China) with written informed consent. All the clinical specimens were snapped-frozen and stored in liquid nitrogen. The ethical approbation was conferred from Committees for Ethical Review in China Pharmaceutical University (Nanjing, China). Pathological diagnosis was made complying with the histology of tumor specimens or biopsy as well as examined by experienced pathologists. The patients’ clinical features are listed in Additional file 1: Table S1. The study was in accordance with all relevant ethical regulations for human research volunteers, and all participants submitted written informed consent.

### Pancreatic cancer cell lines and cell culture

The human pancreatic cancer cell lines (Panc-1, MIApaca-2, SW1990, and BXpc-3), and human pancreatic duct epithelial (HPDE) cells used in the study were obtained from the Type Culture Collection of the Chinese Academy of Sciences (Shanghai, China). The HPDE, Panc-1 and BXpc-3 cells were grown in RPMI-1640 (Cat# 11875093, Gibco); the MIApaca-2 cells were grown in DMEM medium (Cat# 11054020, Gibco), and SW1990 cells were grown in L-15 medium (Cat# 11415064, Gibco). All these mediums were complemented with 10% FBS (Cat# 10099141C, Gibco, USA), 100 U/mL penicillin and 100 ng/mL streptomycin (Cat# C0222, Beyotime, China). The cells were maintained in a humidified chamber supplemented with 5% CO_2_ at 37 °C. The spheroid pancreatic cancer cells were cultured as described in our previous study [11] in DMEM: F12 (Cat# 11765054, Gibco) complemented with 1% FBS, 1% methylcellulose (1500cP, Cat# 9004–67-5, Sigma), 100 ng/ml bFGF (Cat# 500-P18, PeproTech), 20 ng/ml EGF (Cat# 100–47, PeproTech), 2% B27 supplement (Cat# 17504044, Gibco), 1% ITS (5 mg/L insulin, 5 mg/L transferrin, 5 μg/L sodium selenite; Cat# 41400045, Gibco), 100 µg/ml streptomycin (Cat# 15070063, Gibco) and 100 U/ml penicillin under 5% CO_2_ at 37 °C. The 10-day-old spheroids were collected for the following assays.

### RNA isolation, reverse transcription, and quantitative real-time PCR (qRT-PCR)

Total RNA was isolated from pancreatic cancer cells and sample tissues using TRIzol reagent (Cat# 15596018, Invitrogen) and treated with DNase I (Cat# M2181, Promega). Reverse transcription reaction was achieved through usage of PrimeScript™ RT reagent Kit (Cat# RR037A, Takara). Diluted cDNA was employed to conduct qRT-PCR analysis by SYBR Premix Ex Taq II Kit (Cat# DRR041A, Takara) according to the product instruction with the proper primers listed in Additional file 2: Table S2.

### Flow cytometry and cell sorting

Cells were stained using different antibodies conforming to the product instructions, and the used antibodies are provided as Additional file 3: Table S3. A FACSCalibur (BD Immunocytometry Systems) was employed to detect labeled cells or they were sorting with FACS Aria III (BD).

### Plasmid construction and cell transfection

Super-Fidelity DNA Polymerase (Cat# P501-d1, Vazyme) was used to amplify the cDNAs of CTD-3252C9.4, IFI6 and IRF1, which were cloned into the expression vector pcDNA3.1 (Cat# V79020, Invitrogen). All PCR products were verified by DNA sequencing. In Additional file 3: Table S3, primers used for plasmid construction are listed. Cells were transfected with the above plasmids or purchased lentivirus pLVX-CTD-3252C9.4 (General Biosystems) for overexpressing the corresponding genes, and pcDNA3.1 (pcDNA-Ctrl) or pLVX-Ctrl were used as control. The plasmid construct map can be found in Additional file 4: Fig. S1. For knocking down, cells were transfected with Smart Silencers (RiboBio) that contains 3 siRNAs, coincident with si-Ctrl as negative control.

### Proliferation, migration and Invasion assay

Cell proliferation was assayed using Cell Counting Kit 8 (CCK-8) assay, plate colony assay and 5-Ethynyl-2'-deoxyuridine (EdU) assay. CCK-8 and EdU assays were performed using Cell Counting Kit 8 (Cat# CK04, Dojindo) and EdU Cell Proliferation Assay Kit (Cat# C10310-1, RiboBio) base on the kits’ instructions. Plate colony assay was performed as described before [14]. Cell migration and invasion abilities were assessed by transwell assays based on published method [15, 16].

### Apoptosis assay

Annexin V/propidium iodide (PI) staining, TUNEL assay and detection of apoptosis-related proteins (cytochrome-C, Bcl-2, Bax, cytochrome-C, caspase-3, cleaved-caspase-3, caspase-9, cleaved-caspase-9) were applied to estimate cell apoptosis, Annexin V/ PI staining were performed by using Annexin V-FITC/PI Apoptosis Detection Kit (Cat# KGA101, KeyGen Biotech), One-Step TUNEL Apoptosis Assay Kit (Cat# C1089, Beyotime) and Western Blot analysis, respectively.

### Mitochondrial membrane potential detection

The mitochondrial membrane potential (Δψm) was assessed by JC-1 method. Concisely, JC-1 (Cat# C2005, Beyotime) working solutions were used to wash and incubate the indicated cells and this lasted for 20 min at 37 °C in the darkness. The cells were washed using JC-1 wash buffer after incubation and observed by a fluorescence microscope. In healthy cells with a Δψm indicating membrane depolarization, JC-1 forms JC-1 aggregates and shows red fluorescence. Whereas in apoptotic cells, JC-1 remains monomeric and exhibits green fluorescence.

### Xenograft assays in nude mice

We purchased BALB/c nude mice (5–6 weeks old, 18–20 g) from the Model Animal Research Center at Nanjing University (Nanjing, China). They were raised under specific pathogen-free (SPF) conditions at China Pharmaceutical University. All of the animal studies obeyed with Institutional Animal Care and Use Committee (IACUC) regulations. The subcutaneous xenograft mouse model was used to evaluate tumor growth. BALB/c nude mice were randomly divided into two groups (5 mice/group) and accepted subcutaneous injection of different treated Panc-1 cells (1 × 10^6^ suspended in 0.2 mL PBS). The tumor volumes were calculated individually using the formula: length × width ^2^ /2. The mice were sacrificed 27 days after injection, they were injected with sodium pentobarbital (150 mg/kg body weight, Sigma) for euthanasia to minimize suffering and distress according to AVMA Guidelines for the Euthanasia of Animals. The xenograft tumors were harvested and measured. For in vivo metastasis experiments, the mice were randomly divided into two groups (5 mice/group), and different treated Panc-1 cells (1 × 10^6^ suspended in 0.1 mL PBS) were injected into the tail vein of each mouse. Metastases were then examined by bioluminescence images (BLI) by an IVIS Spectrum Xenogen Imaging System (Xenogen) on day 3, 10, 20 and 30. After 30 days, mice were humanely sacrificed by euthanasia. Entire mice livers and lungs tissues were harvested, photographed, and embedded in paraffin for H&E staining and Immunohistochemistry (IHC). The operators and investigators were blinded to the group allocation for all animal experiments. All animal experiments got approvement from the Ethics Committee of China Pharmaceutical University (Permit Number: 2162326).

### Western blot analysis

The proteins were quantified using Bradford Protein Assay Kit (Cat# P0006, Beyotime). Equal amount of denatured protein was separated using 10% SDS-PAGE gels according to the molecular weight. Then, they were transferred to polyvinylidene difluoride membranes, which were immunoblotted with primary antibodies and detected with horseradish peroxidase-conjugated anti-IgG. Antibodies involved in the western blotting are provided in Additional file 3: Table S3. After that, membranes were visualized using Immobilon Western Chemiluminescent HRP Substrate (Cat# WBKLS0050, Millipore) and Image Lab™ Software (Bio-Rad).

### Luciferase reporter assays

The IFI6 promoter region -1337 ~  + 199 bp construct was amplified from genomic DNA of Panc-1 cells. The full length and mutated IFI6 promoter constructs were cloned into the pGL3-basic reporter gene vector and verified by sequencing. The 293 T cells were used for transfection. Following the manufacturer’s instruction, luciferase activities were measured using Dual Luciferase Reporter system (Promega).

### Chromatin immunoprecipitation (ChIP)

ChIP assays were performed using the EZ-ChIP kit (Cat# 17–371, Millipore) according to the instructions. Cells were treated with 1% formaldehyde for cross-link at 37℃ for 10 min. Then, they were sonicated to 300 to 500 bp on average cracking with sodium dodecyl sulfate (SDS) lysis buffer. After incubating with proper antibodies (anti-IRF1) and washing, cross-linked DNA released from the protein-DNA complex and the eluted DNA was further purified and detected by qRT-PCR. The sequences of primers used for ChIP-qPCR are listed in Additional file 2: Table S2.

### RNA pull-down assay

The full length CTD-3252C9.4 sequence was PCR amplified using a T7-containing primer and then reversely transcribed by in vitro Transcription T7 Kit (Cat# 6140, Takara). The targeted RNA was Biotin-labeled with Pierce™ RNA 3’ End Desthiobiotinylation Kit (Cat# 26,103, Thermo). The cells were lysedand streptavidin magnetic beads were used to capture the biotin-labeled CTD-3252C9.4 probe. The biotinylated RNAs were incubated with the protein extract from cells using Pierce™ Magnetic RNA–Protein Pull-Down Kit (Cat# 20,164, Thermo). The protein samples were further detected by Western Blot analysis after the magnetic beads (Cat# G7281, Promega) were eluted. The extracted protein was utilized as a positive control and antisense RNA as a negative control.

### Statistical analysis

Each experiment was performed thrice and data were shown as the mean ± SEM. Statistical analysis was performed using GraphPad Prism 8.0. Student’s t-test was used for two-group comparisons. Pearson correlation analysis was used for analyzing the correlation between groups. *P* < 0.05 was considered statistically significant.

## Results

### CTD-3252C9.4 is down-regulated in pancreatic cancer tissues and cells

**Fig. 1:**
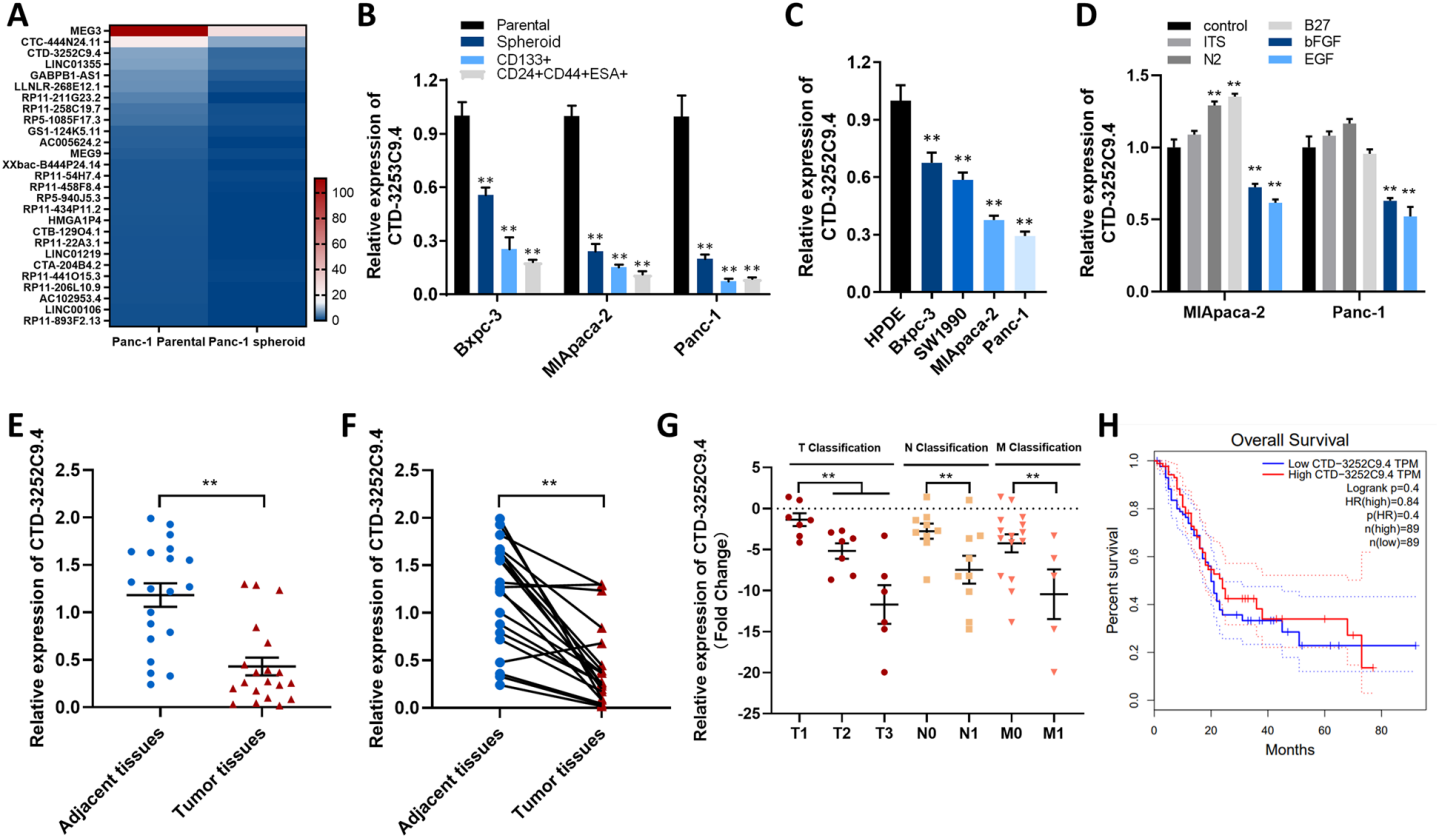
Expression of lncRNA CTD-3252C9.4 in pancreatic cancer cells and tissues. **A** The cluster heat map of all the significantly down-regulated (> 50%) lncRNAs from lncRNA sequencing analysis in Panc-1 spheroid cells compared to Panc-1 parental cells, including CTD-3252C9.4. **B** and **C** Relative expression of CTD-3252C9.4 was detected in PCSCs (**B**) and different pancreatic cancer cells (**C**) by qRT-PCR. **D** Relative expression of CTD-3252C9.4 was detected in MIApaca-2 and Panc-1 cells stimulated by ITS, N2, B27, bFGF and EGF (**E** and **F)**. Relative expression of CTD-3252C9.4 was detected in 20 non-paired (**E**) or paired (**F**) pancreatic cancer tissues and the adjacent normal tissues. **G** Relative expression of CTD-3252C9.4 was detected in pancreatic cancer patients with different T stages, N stages and M stages. **H** The relationship between overall survival (OS) of pancreatic cancer patients and CTD-3252C9.4 expression. All data are shown as the mean ± SEM. *P < 0.05 and **P < 0.01 by two-tailed Student’s *t*-test

Our previous study has shown that several lncRNAs levels were changed remarkably in Panc-1 spheroid cells that are enriched in PCSCs identified by HTS [11]. Among the 27 down-regulated lncRNAs, lncRNA CTD-3252C9.4 was highly ranked (Fig. 1A), which was further verified by qRT-PCR in Panc-1 parental and spheroid cells[11] and in different pancreatic cancer cell lines (Additional file 4: Fig. S2), drew our attention in particular. To assess the relevance of CTD-3252C9.4 in human pancreatic cancer, we first detected the expression of CTD-3252C9.4 in PCSCs and pancreatic cancer cell lines. A novel 3D semi-solid culture system [11] was used to culture Bxpc-3, MIApaca-2 and Panc-1 spheroid cells enriched in either CD133 + or CD24 + CD44 + ESA + PCSCs. Study of Bxpc-3, MIApaca-2 and Panc-1 spheroid cells and PCSCs indicated that the expression of CTD-3252C9.4 in spheroid cells and PCSCs were significantly lower than that in parental pancreatic cancer cells (Fig. 1B). Similarly, we detected the expression levels of CTD-3252C9.4 in different pancreatic cancer cell lines (Panc-1, SW1990, MIApaca-2 and Bxpc-3) and normal human pancreatic duct epithelial (HPDE) cells. We found that CTD-3252C9.4 levels were also lower in pancreatic cancer cells than in normal pancreatic duct epithelial cells (Fig. 1C). ITS (Insulin, Transferrin and Selenium), N2 Supplement, B27 Supplement, Epidermal growth factor (EGF) and b-fibroblast growth factors (b-FGF) were major components added in our sphere-formation pelletizing system, we therefore added these supplements to Panc-1 and MIApaca-2 cells separately and found CTD-3252C9.4 expression was down-regulated by bFGF and EGF, which also exist in the tumor microenvironment (Fig. 1D). Furthermore, the down-regulation of CTD-3252C9.4 mRNA were demonstrated in 20-paired pancreatic cancer tissues and corresponding adjacent normal tissues by qPCR. As shown in Fig. 1E and F, CTD-3252C9.4 expression showed a significant down-regulation in 85% (17 of 20 paired) of the pancreatic cancer tissues. Furthermore, we divided the samples into high (above the median, n = 10) and low (below the median, n = 10) CTD-3252C9.4 expression groups according to the median value of CTD-3252C9.4 levels and then delved the correlation between the expressions of CTD-3252C9.4 and the clinicopathological factors of pancreatic cancer patients (Additional file 1: Table S1). As shown in Fig. 1G, CTD-3252C9.4 level was negatively associated with T (tumor size), N (lymph node) and M (metastasis) classification. We also evaluated the correlation between the expression level of CTD-3252C9.4 and clinical outcomes of pancreatic cancer patients from the TCGA database using GEPIA (Gene Expression Profiling Interactive Analysis). Although no significant difference was shown from the survival rate between the CTD-3252C9.4 high and low expression groups, we found lower expression CTD-3252C9.4 indeed predicted a poorer prognosis in pancreatic cancer patients (Fig. 1H). The above results demonstrated that CTD-3252C9.4 expression is down-regulated in pancreatic cancer.

### CTD-3252C9.4 down-regulation facilitates migration, invasion, proliferation and suppresses apoptosis of pancreatic cancer cells in vitro.

**Fig. 2:**
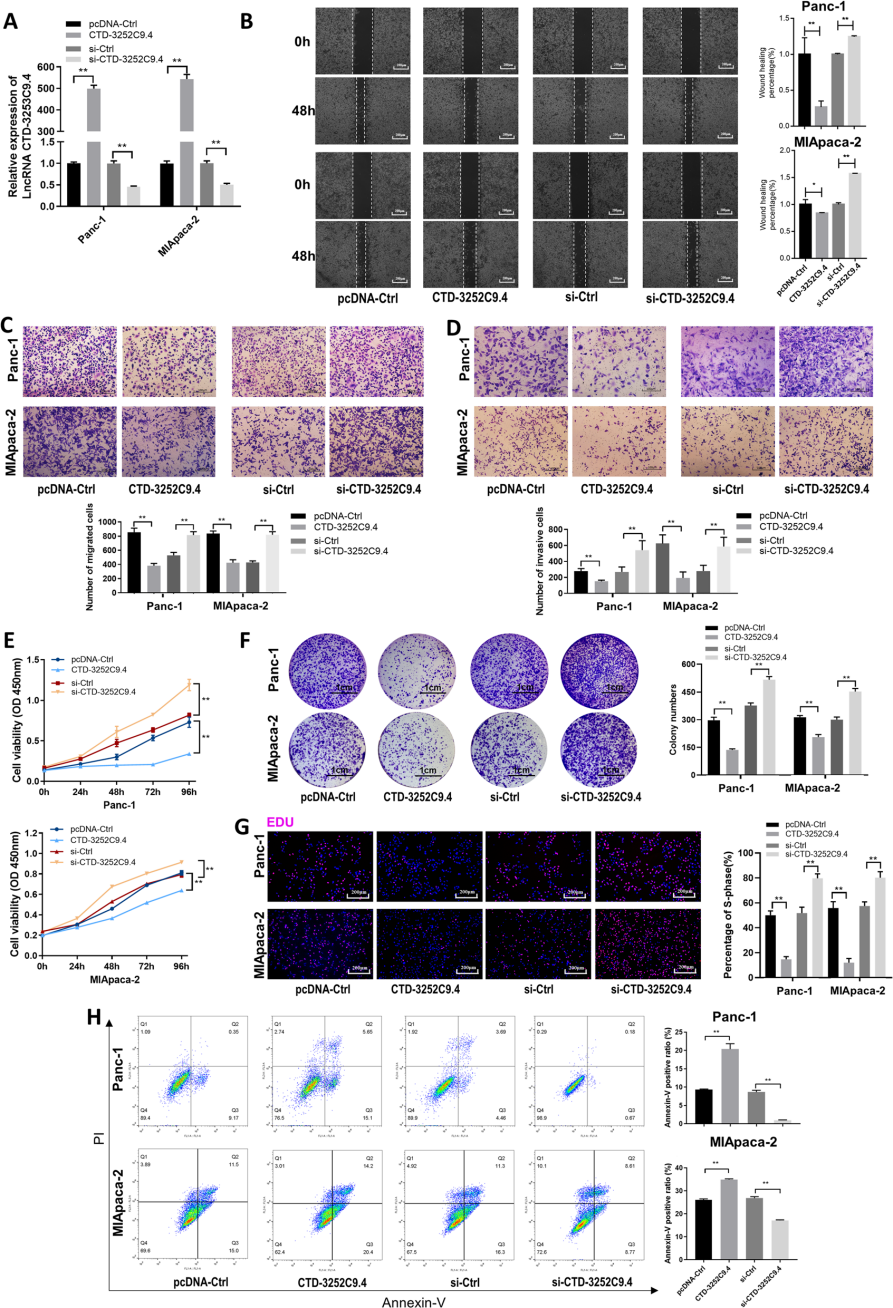
LncRNA CTD-3252C9.4 affects migration, invasion, proliferation and apoptosis of pancreatic cancer cells in vitro. **A** CTD-3252C9.4 overexpression and knockdown efficiencies tested by transfecting CTD-3252C9.4 plasmid and CTD-3252C9.4 siRNAs in Panc-1 and MIApaca-2 cells. **B** Migration of CTD-3252C9.4-overexpressing or CTD-3252C9.4- knocked down Panc-1 and MIApaca-2 cells examined by wound healing assays. Scale bar, 200 μm. Migration (**C**) and invasion (**D**) of CTD-3252C9.4-overexpressing or CTD-3252C9.4- knocked down Panc-1 and MIApaca-2 cells were examined by transwell assays. Scale bar, 100 μm. Proliferation of CTD-3252C9.4-overexpressing or CTD-3252C9.4- knocked down Panc-1 and MIApaca-2 cells was examined by CCK-8 assays (**E**), plate colony assays (**F**, Scale bar, 1 cm) and EdU assays (**G**, Scale bar, 200 μm). **H** Apoptosis of CTD-3252C9.4-overexpressing or CTD-3252C9.4- knocked down Panc-1 and MIApaca-2 cells was examined by Annexin V/ propidium iodide (PI) staining and flow cytometry assays. All data are shown as the mean ± SEM. *P < 0.05 and **P < 0.01 by two-tailed Student’s *t*-test

To explore whether CTD-3252C9.4 was involved in pancreatic cancer progression, we used an overexpression plasmid (CTD-3252C9.4) and an siRNA (si-CTD-3252C9.4) to upregulate or knock down the expression of CTD-3252C9.4 in Panc-1 and MIApaca-2 cells (Fig. 2A). Firstly, we explored the function of CTD-3252C9.4 in regulating pancreatic cancer cell migration and invasion. The wound healing assays (Fig. 2B) and transwell assays (Fig. 2C and D) showed that the migration and invasion capacities of Panc-1 and MIApaca-2 cells were significantly enhanced by depleting CTD-3252C9.4 whereas suppressed by CTD-3252C9.4 overexpression. Secondly, we studied the role of CTD-3252C9.4 in regulating pancreatic cancer cell propagation and apoptosis. The CCK-8 assays, plate colony assays and EdU assays showed that CTD-3252C9.4 depletion significantly promoted cell proliferation, while CTD-3252C9.4 overexpression inhibited the propagative abilities of cells (Fig. 2E-G). Flow cytometric analysis showed that CTD-3252C9.4 overexpression increased the proportion of apoptotic Panc-1 and MIApaca-2 cells, while CTD-3252C9.4 depletion remarkably reduced the apoptotic cell proportion (Fig. 2H). Our findings suggested that CTD-3252C9.4 may function as a tumor suppressor in pancreatic cancer, and the down-regulation of CTD-3252C9.4 contributes to the migration, invasion, proliferation, and anti-apoptosis of pancreatic cancer cells.

### CTD-3252C9.4 inhibits pancreatic tumor growth and metastasis in vivo

**Fig. 3:**
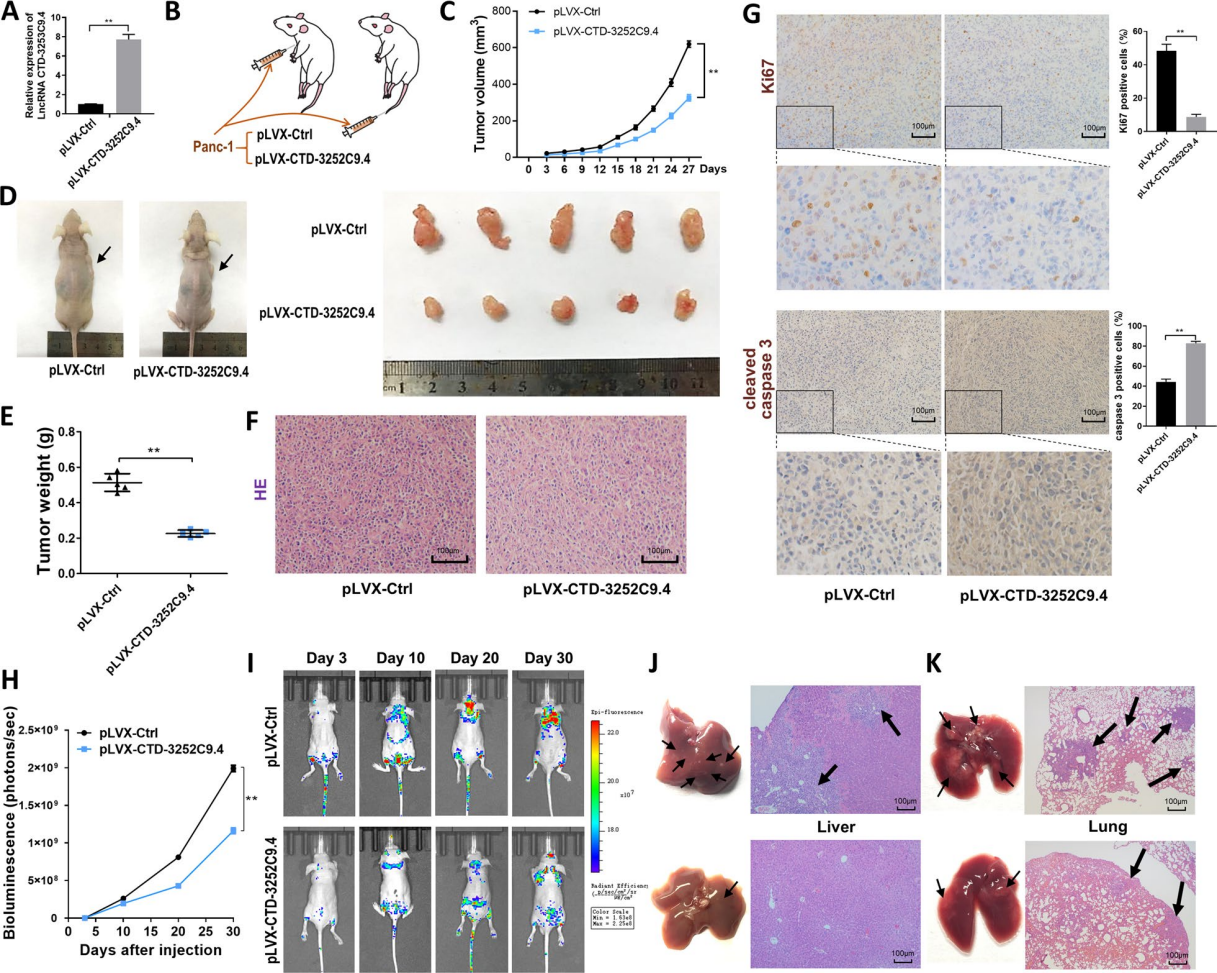
LncRNA CTD-3252C9.4 promotes pancreatic cancer growth and metastasis in vivo. **A** CTD-3252C9.4 overexpression efficiency by transfecting pLVX-CTD-3252C9.4 in Panc-1 cells. **B** Flowchart of the in vivo xenograft experiments designed for examining tumor proliferation and metastasis. The tumor growth of mice subcutaneously implanted with the indicated Panc-1 cells. Tumor volume (**C**) and weight (**E**) were measured, and tumor size is shown in picture (**D**). Representative H&E staining (**F**), Ki-67 and cleaved caspase 3 immunohistochemical staining (**G**) of the tumor sections. Quantitative analysis of the fluorescence intensities (**H**) and representative BLI images (**I**) of mice intravenously injected with the indicated Panc-1 cells on days 3, 10, 20, and 30 after injection. The gross lesions (left) and representative H&E-stained sections (right) of liver (**J**) and lung (**K**) tissues isolated from the intravenously injected mice. Black arrows indicate the metastatic nodules. Scale bar, 100 μm. All data are shown as the mean ± SEM. *P < 0.05 and **P < 0.01 by two-tailed Student’s *t*-test

We next evaluated the effects of CTD-3252C9.4 on the growth and metastasis of Panc-1 cell xenografts in mice. We transfected Panc-1 cells with control (pLVX-Ctrl) or CTD-3252C9.4-overexpressing (pLVX-CTD-3252C9.4) lentivirus (Fig. 3A). As shown in Fig. 3B, we subcutaneously implanted different stable Panc-1 cells into nude mice via the armpit to test the anti-proliferative effect of CTD-3252C9.4, or intravenously injected different stable Panc-1 cells into nude mice via the tail vein to evaluate the effect of CTD-3252C9.4 on tumor metastasis. We assessed tumor growth every 3 days after implantation (Fig. 3C). The tumors were harvested and assessed after all mice were euthanized on day 27. A significant reduction was observed in the size and weight of the tumors in the CTD-3252C9.4-overexpressing xenografts (Fig. 3D and E) compared with control group. Additionally, H&E staining showed that the histopathology of tumors formed in the CTD-3252C9.4-overexpressing xenografts exhibited decreased cell mitosis (Fig. 3F). The cell proliferative activity, which was assessed via the Ki-67 + tumor cell percentage, also declined in tumors from the CTD-3252C9.4-overexpressing xenografts. Meanwhile, CTD-3252C9.4-overexpressing tumors showed increased expression level of cleaved caspase-3 compared with control group (Fig. 3G). These results indicated that overexpression of CTD-3252C9.4 can significantly repress tumor growth in vivo through inhibiting cell proliferation and promoting apoptosis. From day 3 to day 30 post-tail vein injection, BLI were taken at intervals. After analysis of the BLI scans, it was revealed that CTD-3252C9.4 overexpression in Panc-1 cells caused reduced tumor metastasis in lung and liver (Fig. 3H and I). Compared to the control, the CTD-3252C9.4 overexpression group was observed with reduced metastatic lesions both at the surface of the lungs and livers. H&E staining showed that CTD-3252C9.4 overexpression significantly reduced the numbers and the areas of metastatic nodules in the lungs and livers of mice (Fig. 3J and K). The above data supported that CTD-3252C9.4 could inhibit pancreatic cancer cell metastasis.

### IFI6 is a crucial target of CTD-3252C9.4 to exert function in pancreatic tumorigenesis

**Fig. 4:**
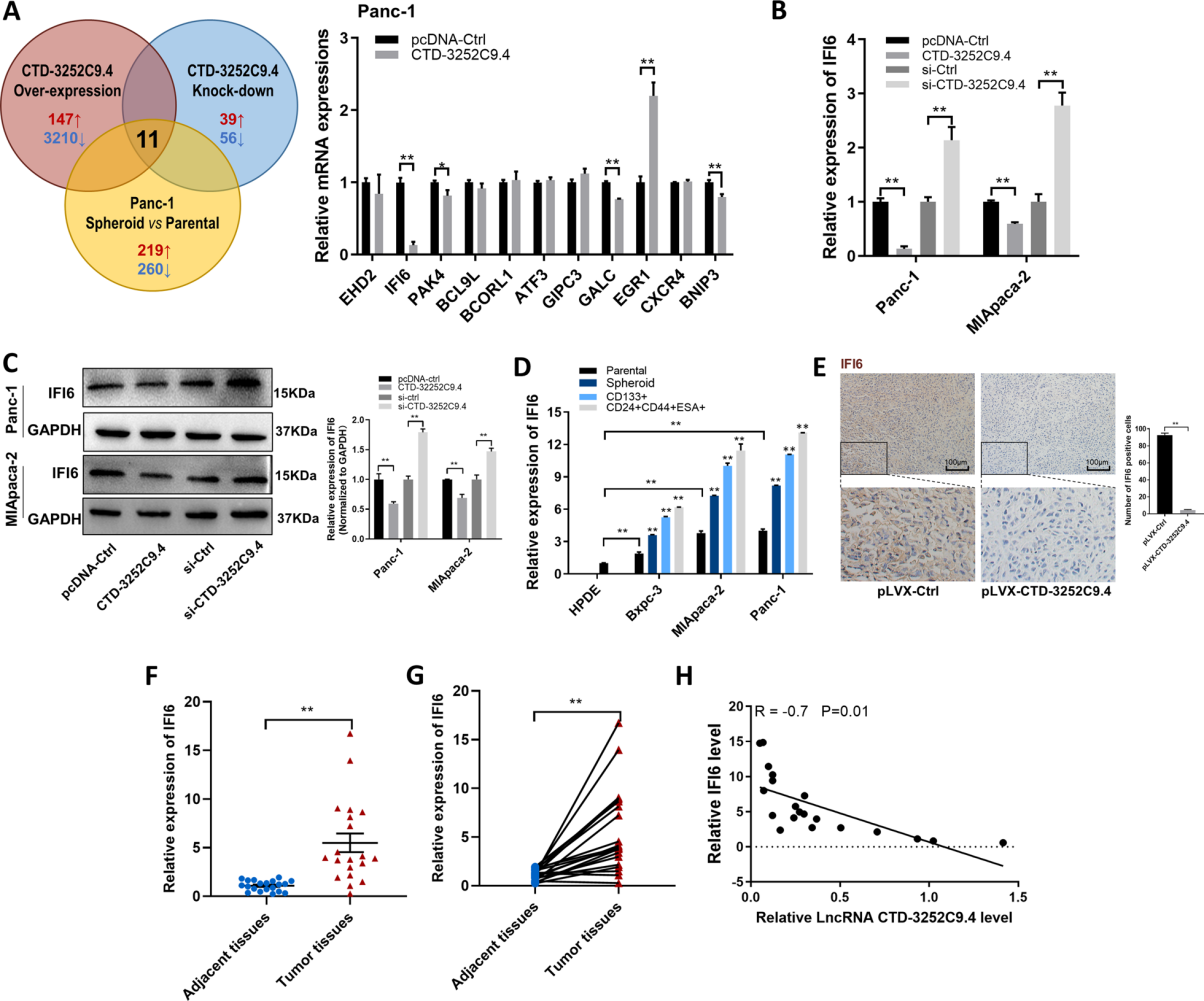
LncRNA CTD-3252C9.4 down-regulates IFI6 expression in pancreatic cancer cell lines. **A** Left: Venn diagram analysis of three independent HTS reveals 11 shared potential targets of CTD-3252C9.4. Right: 11 potential target genes were detected in CTD-3252C9.4-overexpressing Panc-1 cells. Relative IFI6 mRNA (**B**) and protein (**C**) levels in CTD-3252C9.4-overexpressing or CTD-3252C9.4-knocked down Panc-1 and MIApaca-2 cells. **D** Relative expression of IFI6 was detected in different pancreatic cancer cells and PCSCs. **E** Representative images of IFI6 immunohistochemical staining of the tumor sections. Relative expression of IFI6 was detected in 20 non-paired (**F**) or paired (**G**) pancreatic cancer tissues and the adjacent normal tissues. **H** Spearman correlation analysis of the fold changes of CTD-3252C9.4 and IFI6 mRNA in 20 paired pancreatic cancer tissues and the adjacent normal tissues. All data are shown as the mean ± SEM. *P < 0.05 and **P < 0.01 by two-tailed Student’s *t*-test

According to the previous study, Das et. al found that gain or loss of CTD-3252C9.4 perturbed the expression of a large number of genes in HEK293T cells detected by RNA-Seq [12]. Combined with our previous study[11], 11 genes were found changed in CTD-3252C9.4 up- or down-regulated HEK293T and Panc-1 spheroid cells compared with their control. To identify the potential target of CTD-3252C9.4 in pancreatic cancer cells, we examined the expression of above 11 genes in CTD-3252C9.4-overexpressing Panc-1 cells. As shown in Fig. 4A, the expression of IFI6, an anti-apoptotic and pro-proliferative gene, had a remarkable reduction, which particularly drew our attention. In order to confirm that IFI6 was the downstream target of CTD-3252C9.4, we detected the mRNA and protein levels of IFI6 in CTD-3252C9.4-overexpressing or CTD-3252C9.4-knocked down Panc-1 and MIApaca-2 cells. In both pancreatic cancer cell lines, we found the opposite expression patterns of CTD-3252C9.4 and IFI6 (Fig. 4B and C). The negative correlation between CTD-3252C9.4 and IFI6 expression levels was further verified in different pancreatic cancer cell lines and PCSCs, CTD-3252C9.4-overexpressing tumors, 20-paired pancreatic cancer tissues and corresponding adjacent normal tissues (Fig. 4D-H).

**Fig. 5:**
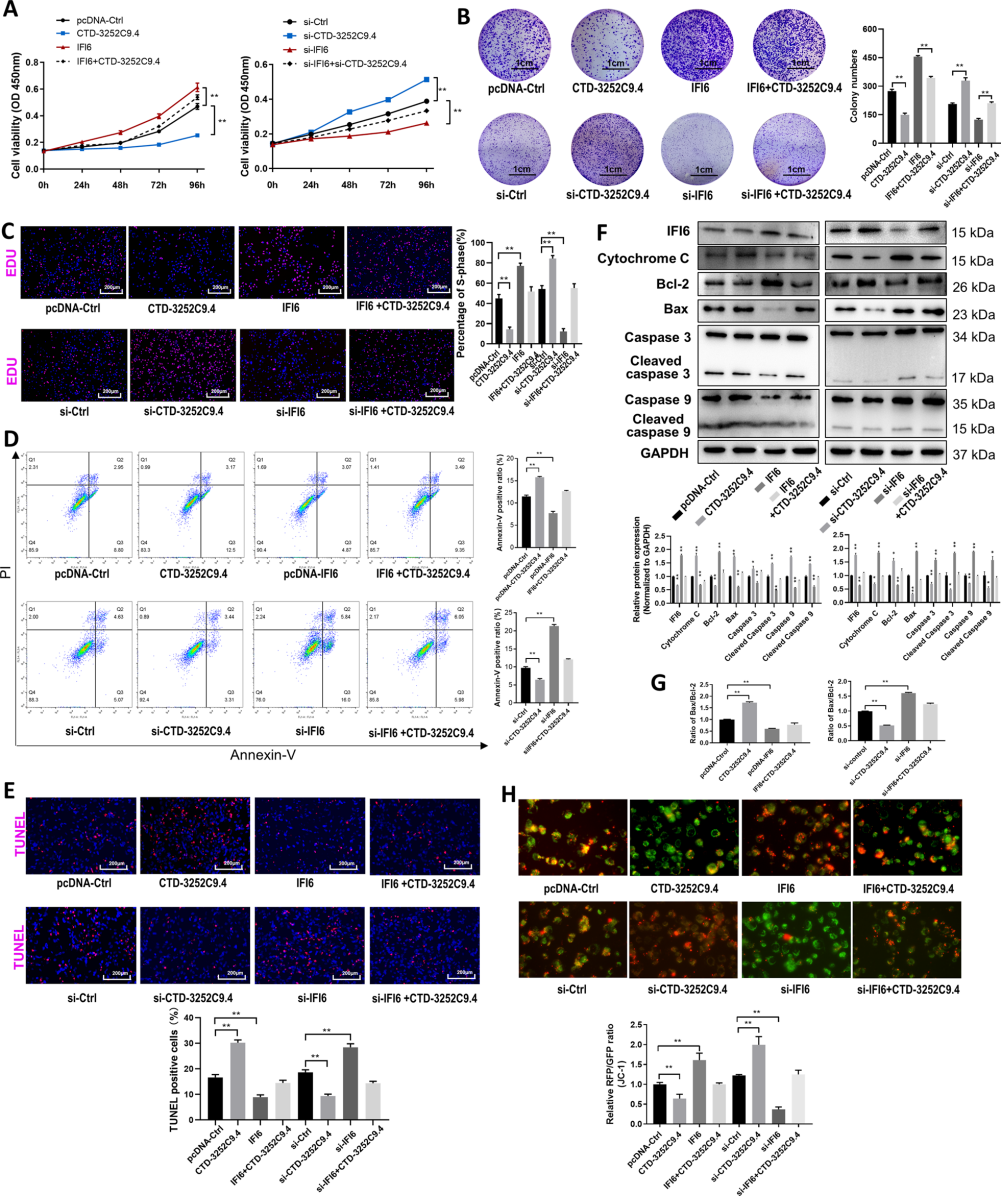
LncRNA CTD-3252C9.4 regulates pancreatic cancer cells migration, invasion, proliferation and apoptosis via targeting IFI6. Proliferation of Panc-1 cells transfected with indicated plasmids was detected by CCK-8 assays (**A**), plate colony assays (**B**, Scale bar, 1 cm) and EdU as says (**C**, Scale bar, 200 μm). Apoptosis of Panc-1 cells transfected with indicated plasmids was detected by Annexin V/ propidium iodide (PI) staining and flow cytometry assays (**D**) and TUNEL assay (**E**). Relative expression of apoptosis-related proteins was detected in cells above (**F**). Bcl-2/Bax ratio was determined by comparing the relative expression levels of Bcl-2 and Bax (**G**). **H** Detection of mitochondrial membrane potential by JC-1 staining in Panc-1 cells transfected with indicated plasmids. All data are shown as the mean ± SEM. *P < 0.05 and **P < 0.01 by two-tailed Student’s *t*-test

To prove that the anti-proliferative and pro-apoptotic effects of CTD-3252C9.4 were mediated by down-regulating IFI6, we conducted the rescue experiments. Firstly, we observed that overexpression of IFI6 promoted while knockdown of IFI6 repressed Panc-1 cell proliferation. IFI6 overexpression or down-regulation also attenuated the effects of CTD-3252C9.4 overexpression or down-regulation on Panc-1 cell proliferation (Fig. 5A–C). Secondly, transfection of IFI6 vector not only suppressed the apoptosis of Panc-1 cells, but also successfully mitigated CTD-3252C9.4 overexpression-induced Panc-1 cell apoptosis, while transfection of IFI6 siRNA displayed the inverse effects (Fig. 5D–G). Finally, to investigate whether CTD-3252C9.4 targeting IFI6 could affect cell survival through regulating the function of mitochondria, we applied a usual method to detect apoptosis mediated by mitochondrial as well as mitochondrial dysfunction, through usage of the mitochondrial membrane potential indicator JC-1. Consistent with the above results, CTD-3252C9.4 overexpression led to a significant reduction in aggregate (red) and an enlargement in monomer forms (green), which indicate a mitochondria-dependent apoptosis. However, IFI6 overexpression obstructed the loss of aggregate and the increment of the monomer forms. On the contrary, knockdown of CTD-3252C9.4 increased the ratio between aggregate and monomer forms while IFI6 knockdown caused a decrease in the ratio of aggregate to monomer (Fig. 5H). Overall, these results indicated that IFI6 is a crucial target for the lncRNA CTD-3252C9.4 to exert biological roles in pancreatic cancer.

### CTD-3252C9.4 represses IFI6 expression through binding with its transcription factor IRF1

**Fig. 6:**
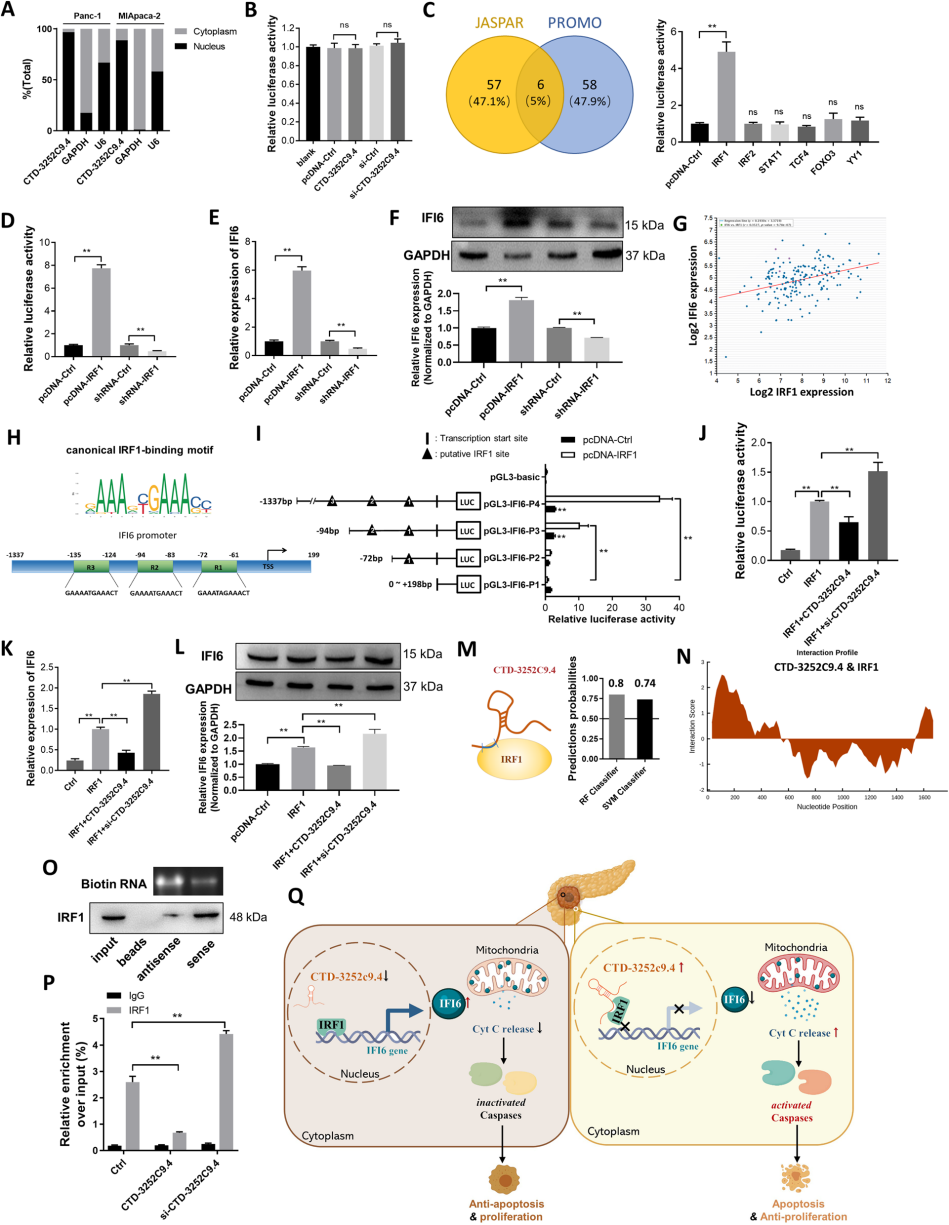
LncRNA CTD-3252C9.4 inhibits IFI6 expression through binding with its transcription factor IRF1. **A** Cytoplasm and nuclear distribution of CTD-3252C9.4 in pancreatic cancer cells detected by fractionation of Panc-1 and MIApaca-2 cells followed by qRT-PCR. U6 and GAPDH RNAs served as the positive control for nuclear gene expression and cytoplasmic gene expression, respectively. **B** Luciferase activity was measured in Panc-1 cells co-transfected with pcDNA-Ctrl, pcDNA-CTD-3252C9.4, si-Ctrl, or si-CTD-3252C9.4 and PGL3-IFI6 vectors which included IFI6 promoter. **C** Left: 11 potential transcription factors of IFI6 were predicted by both JASPAR and PROMO database. Right: Luciferase activity was measured in Panc-1 cells co-transfected with PGL3-IFI6 vectors and vectors expressing the above 11 genes. **D** Luciferase activity was measured in Panc-1 cells co-transfected with pcDNA-Ctrl, pcDNA-IRF1, si-Ctrl, or si- IRF1 and PGL3-IFI6 vectors. Relative IFI6 mRNA (**E**) and protein (**F**) levels in IRF1-overexpressing or IRF1-knocked down Panc-1 cells. **G** Spearman correlation analysis of the fold change of IFI6 mRNA and IRF1 mRNA in 1104 human breast cancer tissues in starBase public database from TCGA project. **H** Putative IRF1 binding sites on the promoter region of IFI6. **I** Mutagenesis in the putative binding site of − 72 ~  − 61 bp fragment of the IFI6 promoter abrogated the induction activity of RUNX2 in the Panc-1 cells. **J** Luciferase activity was measured in Panc-1 cells co-transfected with pcDNA-Ctrl plus si-Ctrl, pcDNA-IRF1 plus si-Ctrl, pcDNA-IRF1 plus pcDNA-CTD-3252C9.4, or pcDNA-IRF1 plus si-CTD-3252C9.4 and PGL3-IFI6 vectors. Relative IFI6 mRNA (**K**) and protein (**L**) levels in Panc-1 cells transfected with pcDNA-Ctrl plus si-Ctrl, pcDNA-IRF1 plus si-Ctrl, pcDNA-IRF1 plus pcDNA-CTD-3252C9.4, or pcDNA-IRF1 plus si-CTD-3252C9.4. **M** The possibility of interaction between CTD-3252C9.4 and IRF1 was predicted by RPISeq predictions. **N** CatRAPID fragments module prediction of the interaction profile and matrix between CTD-3252C9.4 and IRF1. **O** The interaction between CTD-3252C9.4 and IRF1 were verified by RNA pulldown assays. **P** The enrichment of IRF1 on the IFI6 promoter relative to IgG in control, CTD-3252C9.4-overexpressing or CTD-3252C9.4- knocked down Panc-1 cells detected by ChIP-qPCR assays. **Q** A working model for the role of IRF1-targeted CTD-3252C9.4 in pancreatic cancer proliferation and apoptosis. During pancreatic tumorigenesis, silencing of CTD-3252C9.4 facilitated proliferation and inhibited apoptosis of pancreatic cancer cells through up-regulating the expression of IFI6 via binding with its transcription factor IRF1. All data are shown as the mean ± SEM. *P < 0.05 and **P < 0.01 by two-tailed Student’s t-test

To elucidate the molecular mechanisms by which CTD-3252C9.4 inhibits IFI6 expression, we first studied the cellular localization of CTD-3252C9.4 in pancreatic cancer cells. Through cellular fractionation assays (Fig. 6A), we found that CTD-3252C9.4 was mainly localized in the nucleus of Panc-1 and MIApaca-2 cells, which suggested that CTD-3252C9.4 might regulate IFI6 expression at the transcriptional level. Next, we determined whether CTD-3252C9.4 directly affected IFI6 transcription through binding with its promoter. Luciferase reporter assays showed that neither CTD-3252C9.4 overexpression nor knockdown could affect the luciferase activity by binding to the promoter region of IFI6 (Fig. 6B). As nuclear lncRNAs can bind to transcription factors and regulate their transcriptional activity, we further questioned whether CTD-3252C9.4 regulated IFI6 transcription through interacting with its transcription factors. JASPAR and PROMO databases were used to predict the transcription factors binding to the IFI6 promoter (Fig. 6C), and we found overexpression or knockdown of IRF1, a transcriptional activator belonging to the IRF family of transcription factors, could regulate the transcription of IFI6 (Fig. 6D–F). In 1104 breast cancer tissues (Fig. 6G; StarBase database), the positive correlation between IRF1 and IFI6 mRNA levels was supported by Spearman correlation analysis. Additionally, the binding sites between IRF1 and IFI6 promoter were identified. Sequence analyses of the IFI6 promoter region showed putative IRF1-binding sites (Fig. 6H). Luciferase reporter assays showed that IRF1 significantly enhanced the luciferase activity level by binding to a promoter region − 72 ~  − 61 bp upstream of the transcription start point of IFI6 (Fig. 6I). Furthermore, we found overexpression of CTD-3252C9.4 abrogated the upregulation of IFI6 expression activated by IRF1, while knockdown of CTD-3252C9.4 enhanced the IRF1-activating IFI6 overexpression (Fig. 6J-L). RPISeq predictions showed RF and SVM of CTD-3252C9.4-IRF1 were 0.8 and 0.74 (Fig. 6M). It is revealed by CatRAPID fragments that the 59–235 nucleotide positions of the CTD-3252C9.4 sequence might bind to the IRF1 protein with high propensities (Fig. 6N). Moreover, in the biotin-labeled sense CTD-3252C9.4 group, IRF1 was detected by RNA pulldown assays (Fig. 6O). The ChIP assays also demonstrated that overexpression of CTD-3252C9.4 decreased the binding of IRF1 at IFI6 promoter region, while knockdown of CTD-3252C9.4 increased the binding level (Fig. 6P). Taken together, the results suggested that CTD-3252C9.4 could block the binding of IRF1 to the promoter region of IFI6, which led to the transcriptional inhibition of IFI6.

## Discussion

In the current study, we illuminated the pivotal role of CTD-3252C9.4 in pancreatic cancer and its underlying mechanisms. We identified that CTD-3252C9.4 was down-regulated in clinical pancreatic cancer samples and cell lines. In vitro and in vivo functional experiments showed that CTD-3252C9.4 controls the growth, apoptosis and metastasis of pancreatic cancer cells. Mechanistically, we showed that the function of CTD-3252C9.4 was mediated by IFI6, a regulator of cell apoptosis and survival. By binding to IRF1 to abolish its transcriptional activity, CTD-3252C9.4 inhibits the transcription of IFI6, which then leads to the activation of apoptosis signaling pathway in pancreatic cancer cells. This mechanism is presented diagrammatically in Fig. 6Q.

Numerous studies have validated the crucial effects of aberrantly expressed lncRNAs on the course of multiple cancers, including pancreatic cancer. Nevertheless, the functions of most lncRNAs, especially novel lncRNAs, are still unknown. CTD-3252C9.4 is a 1,774-nt, nuclear localized and chromatin associated lncRNA that located on 19p13.12 (chr19:13,823,880–13,842,928) with few studies. Recent reports indicated that CTD-3252C9.4 is abnormally expressed in multiple cancers. It is found highly expressed in head and neck cancers, hepatocellular carcinoma, colon and ovary cancer [13, 17], while significantly reduced in prostate, uterus, breast, and kidney cancers[12], which indicated that CTD-3252C9.4 has diverse expression patterns in different cancers. The roles of CTD-3252C9.4 in nasopharyngeal and hepatocellular carcinomas have been revealed. The upregulation of CTD-3252C9.4 in nasopharyngeal carcinoma was associated with poor prognosis and promoted the migration and invasion capacity of nasopharyngeal carcinoma cells via affecting the expression of cytoskeletal and adhesion-related molecules [18]. Increased CTD-3252C9.4 expression was markedly associated with aggressive clinicopathological factors and shorter survival time in patients with hepatocellular carcinoma. Through binding with EZH2 to inhibit E-cadherin expression, CTD-3252C9.4 contributes to hepatocellular carcinoma cell invasion and migration [17]. The expression and function of CTD-3252C9.4 in pancreatic cancer, however, have not been reported so far. The molecular underlying mechanisms of CTD-3252C9.4 are still poorly understood. Herein, we showed that CTD-3252C9.4 was down-regulated in pancreatic cancer, and it was correlated negatively with T stage, N stage and M stage of pancreatic cancer patients, which suggested the potential tumor suppressor role of CTD-3252C9.4 in pancreatic cancer. Overexpression of CTD-3252C9.4 repressed the migration, invasion and proliferation of pancreatic cancer cells while promoted the cell apoptosis. Thus, down-regulation of CTD-3252C9.4 contributed to the progression of pancreatic cancer.

Mechanistically, CTD-3252C9.4 was identified as a decoy lncRNA to exert its function in the present study. Decoy lncRNAs repress transcription in an indirect way by binding regulatory factors such as transcription factors, chromatin remodelers, or other RNA-binding proteins, thus sequestering them from their specific target sites [19]. We found that CTD-3252C9.4 prevents transcriptional factor IRF1 from binding to the promoter region of IFI6 through combining with IRF1, in turn represses IFI6 transcription. It has been shown that IFI6 plays anti-apoptosis roles in gastric and breast cancer cells, human myeloma cells and vascular endothelia cells, mainly through stabilizing mitochondrial membrane potential, inhibiting activation of caspase 3 and caspase 9, and decreasing Bax/Bcl-2 [5, 6, 20]. Since anti-apoptosis plays a crucial role for tumor development and the functional role of IFI6 in pancreatic cancer has not been clarified as yet, we focused predominantly on the effects of CTD-3252C9.4 targeting IFI6 on pancreatic cancer cell apoptosis and survival. Here, we firstly verified that IFI6 expression was upregulated and negatively correlated with CTD-3252C9.4 expression in pancreatic cancer cells, tissues, and tumors from mice. Also, IFI6 exhibited opposite effects on cell proliferation and apoptosis compared to CTD-3252C9.4. Then, rescue assays indicated that the impaired pancreatic cancer cells growth and facilitating apoptosis triggered by overexpression of CTD-3252C9.4 could be retrieved by up-regulation of IFI6.

## Conclusion

Taken together, we discovered that down-regulation of CTD-3252C9.4 facilitates pancreatic cancer progression via unbinding IRF1 to amplify IFI6 expression, hindering cell apoptosis and facilitating cell proliferation. Our study demonstrated that CTD-3252C9.4/IRF1/IFI6 axis may be a novel therapeutic target in pancreatic cancer.

## Supplementary Information

**Additional file 1: Table S1.** CTD-3252C9.4 expression and clinicopathological features in 40 patients with pancreatic ductal carcinoma (PADC).

**Additional file 2: Table S2.** Sequences of primers used for qRT-PCR, plasmid construction and ChIP-qPCR.

**Additional file 3: Table S3.** Antibodies used for western blotting (WB), immunoprecipitation (IP) and flow cytometry (FC).

**Additional file 5: Fig. S1.** Plasmid map. (A-F) Plasmid maps for pcDNA-Ctrl (A), pcDNA-CTD-3252C9.4 (B), pcDNA-IFI6 (C), pcDNA-IRF1 (D), pLVX-Ctrl (E) and pLVX- CTD-3252C9.4 (F).** Fig. S2.** Relative expression of 10 lncRNAs down-regulated in Panc-1 spheroid cells compared with parental cells were detected in different pancreatic cancer cells.

## Data Availability

Not applicable.

## Abbreviations

PC: Pancreatic cancer
lncRNAs: Long non-coding RNAs
mRNAs: Messenger RNAs
RT-qPCR: Real-time quantitative polymerase chain reaction
CCK-8: Cell counting kit-8
EdU: 5-Ethynyl-2′-deoxyuridine
IFI6: Interferon alpha inducible protein 6
HTS: High-throughput sequencing
PCSCs: Pancreatic cancer stem cells
IRF1: Interferon regulatory factor 1
HPDE: Human pancreatic duct epithelial
PI: Propidium iodide
IACUC: Institutional Animal Care and Use Committee
ITS: Insulin, Transferrin and Selenium
EGF: Epidermal growth factor
b-FGF: B-Fibroblast growth factors
SPF: Specific pathogen-free
BLI: Bioluminescence images
ChIP: Chromatin immunoprecipitation
GEPIA: Gene Expression Profiling Interactive Analysis
