# Supplementary material for: LncRNA CTD-3252C9.4 modulates pancreatic cancer cell survival and apoptosis through regulating IFI6 transcription
Source: Cancer Cell Int. 2021 Aug 16;21:433. doi: 10.1186/s12935-021-02142-0 (PMC8365976; doi:10.1186/s12935-021-02142-0)
Supplement: Supplementary file 1 — Additional file 1: Table S1. CTD-3252C9.4 expression and clinicopathological features in 40 patients with pancreatic ductal carcinoma (PADC). [file 12935_2021_2142_MOESM1_ESM.docx]

| **Table S1. CTD-3252C9.4 expression and clinicopathological features in 40 patients with pancreatic ductal carcinoma (PADC).** | | | |
| --- | --- | --- | --- |
| **Characteristics** | **Expression of CTD-3252C9.4** | | ***p* value*** |
|  | **low** | **high** |  |
| **Sex** |  |  | 0.639 |
| male | 6 | 7 |  |
| female | 4 | 3 |  |
| **Age** |  |  | 0.653 |
| ≤60 | 5 | 6 |  |
| >60 | 5 | 4 |  |
| **Tumor Differentiation** |  |  | 0.608 |
| Well-differentiated | 0 | 4 |  |
| Moderately differentiated | 3 | 3 |  |
| Poorly differentiated | 7 | 3 |  |
| **T Classification** |  |  | 0.041* |
| T1 | 1 | 6 |  |
| T2 | 4 | 3 |  |
| T3 | 5 | 1 |  |
| **N Classification** |  |  | 0.056 |
| N0 | 2 | 7 |  |
| N1 | 6 | 3 |  |
| N2 | 2 | 0 |  |
| **TNM stage** |  |  | 0.114 |
| Ⅰ/Ⅱ | 5 | 9 |  |
| Ⅲ/Ⅳ | 5 | 1 |  |
| Median expression level was used as a cutoff to divide the 20 patients into CTD-3252C9.4 low group (n = 10) and CTD-3252C9.4 high group (n =10). Two-sided χ2 test. * p<0.05 | | | |
