# Supplementary material for: LncRNA CTD-3252C9.4 modulates pancreatic cancer cell survival and apoptosis through regulating IFI6 transcription
Source: Cancer Cell Int. 2021 Aug 16;21:433. doi: 10.1186/s12935-021-02142-0 (PMC8365976; doi:10.1186/s12935-021-02142-0)
Supplement: Supplementary file 2 — Additional file 2: Table S2. Sequences of primers used for qRT-PCR, plasmid construction and ChIP-qPCR. [file 12935_2021_2142_MOESM2_ESM.docx]

**Table S2. Sequences of primers used for qRT-PCR, plasmid construction and ChIP-qPCR**

| **Primer names** | **Sequences (from 5’ to 3’)** | |
| --- | --- | --- |
| **Sequences of primers used for qRT-PCR** |  | |
| CTD-3252C9.4 forward | GAGGAGGCACACTGAAGTCC | |
| CTD-3252C9.4 reverse | CCAAGCTTCTGTCCACGACT | |
| GAPDH forward | GGAGCGAGATCCCTCCAAAAT | |
| GAPDH reverse | GGCTGTTGTCATACTTCTCATGG | |
| EDH2 forward | TCCGCAAACTCAACCCTTTC | |
| EDH2 reverse | TCTCCAGGACCTGATTAGGGA | |
| IFI6 forward | GGTCTGCGATCCTGAATGGG | |
| IFI6 reverse | TCACTATCGAGATACTTGTGGGT | |
| PAK4 forward | ATCTGGTCGCTGGGGATAATG | |
| PAK4 reverse | CAGGTTGTCCCGAATCATCTTC | |
| BCL9L forward | CTTGAAGTCGCCCACCCTTAG | |
| BCL9L reverse | GCCGTCTTAGGTGAGGCAA | |
| BCORL1 forward | GGACTCCACTCAACACCCAAA | |
| BCORL1 reverse | CCAGTTCCCAGAGTTGCCAAA | |
| ATF3 forward | CCTCTGCGCTGGAATCAGTC | |
| ATF3 reverse | TTCTTTCTCGTCGCCTCTTTTT | |
| GIPC3 forward | CAAGAGGGCCTTCGATATGATTG | |
| GIPC3 reverse | ATGCCCATGTAGCTTTCCAGC | |
| GALC forward | GCCAAGCGTTACCATGATTTG | |
| GALC reverse | CCACCTTGAAGAGTTCGGCA | |
| EGR1 forward | ACCCCTCTGTCTACTATTAAGGC | |
| EGR1 reverse | TGGGACTGGTAGCTGGTATTG | |
| CXCR4 forward | ACTACACCGAGGAAATGGGCT | |
| CXCR4 reverse | CCCACAATGCCAGTTAAGAAGA | |
| BNIP3 forward | TGAGTCTGGACGGAGTAGCTC | |
| BNIP3 reverse | CCCTGTTGGTATCTTGTGGTGT | |
| IRF1 forward | CTGTGCGAGTGTACCGGATG | |
| IRF1 reverse | ATCCCCACATGACTTCCTCTT | |
| **Sequences of primers used for plasmid construction** | |  |
| pcDNA3.1-CTD-3252C9.4 forward | CGCGGATCCGGGGTCAAGCCCCCTTGGA | |
| pcDNA3.1-CTD-3252C9.4 reverse | CCGCTCGAGTGAAGTCAGGGCAACTTTTATTTAC | |
| pcDNA3.1-IFI6 forward | CCCAAGCTTATGCGGCAGAAGGCGGTAT | |
| pcDNA3.1-IFI6 reverse | CCGGAATTCCTACTCCTCATCCTCCTCACTA | |
| pcDNA3.1-IRF1 forward | TCTTAGCATCTCGGCTGGACTTC | |
| pcDNA3.1-IRF1 reverse | CGATACAAAGCAGGGGAAAAGG | |
| **Sequences of primers used for ChIP-qPCR** |  | |
| IFI6 promoter forward | TGTTCCCGCTGGGCGGAGCT | |
| IFI6 promoter reverse | CGTCTCACCCTAGGCAGCCA | |
