# Supplementary material for: LncRNA CTD-3252C9.4 modulates pancreatic cancer cell survival and apoptosis through regulating IFI6 transcription
Source: Cancer Cell Int. 2021 Aug 16;21:433. doi: 10.1186/s12935-021-02142-0 (PMC8365976; doi:10.1186/s12935-021-02142-0)
Supplement: Supplementary file 3 — Additional file 3: Table S3. Antibodies used for western blotting (WB), immunoprecipitation (IP) and flow cytometry (FC). [file 12935_2021_2142_MOESM3_ESM.docx]

**Table S3. Antibodies used for western blotting (WB), immunoprecipitation (IP) and flow cytometry (FC).**

| **Protein** | **Application** | **Antibody** | **Origin** | **dilution** | **Molecular weight** |
| --- | --- | --- | --- | --- | --- |
| GAPDH | WB | D16H11, Cell Signaling Technology | Rabbit | 1:1000 | 37 KD |
| IFI6 | WB | A6157, ABclonal | Rabbit | 1:1000 | 15KD |
| Cytochrome C | WB | A0225, ABclonal | Rabbit | 1:1000 | 15KD |
| Bax | WB | A7626, ABclonal | Rabbit | 1:1000 | 23KD |
| Bcl-2 | WB | A0208, ABclonal | Rabbit | 1:1000 | 26KD |
| Caspase 9/  cleaved caspase 9 | WB, IHC, IF | A2636, ABclonal | Rabbit | 1:1000 | 35/15KD |
| Caspase 3/  cleaved caspase 3 | WB, IHC, IF | A0214, ABclonal | Mouse | 1:1000 | 34/17KD |
| IRF1 | WB, IHC, IF | A7692, ABclonal | Mouse | 1:1000 | 48kD |
| IgG | CHIP, RIP | ab18413, Abcam | Mouse | 1:10 | 150kD |
| CD44 | FC | 559942, BD Pharmingen™ | Mouse | 1:200 | 81KD |
| CD24 | FC | 555428, BD Pharmingen™ | Mouse | 1:200 | 9KD |
| ESA | FC | 25-9326-42, eBioscience | Mouse | 1:100 | 35KD |
| CD133 | FC | 566596, BD Pharmingen™ | Mouse | 1:200 | 97KD |
