# Supplementary figures and images for: LncRNA CTD-3252C9.4 modulates pancreatic cancer cell survival and apoptosis through regulating IFI6 transcription
Source: Cancer Cell Int. 2021 Aug 16;21:433. doi: 10.1186/s12935-021-02142-0 (PMC8365976; doi:10.1186/s12935-021-02142-0)

Figure S1

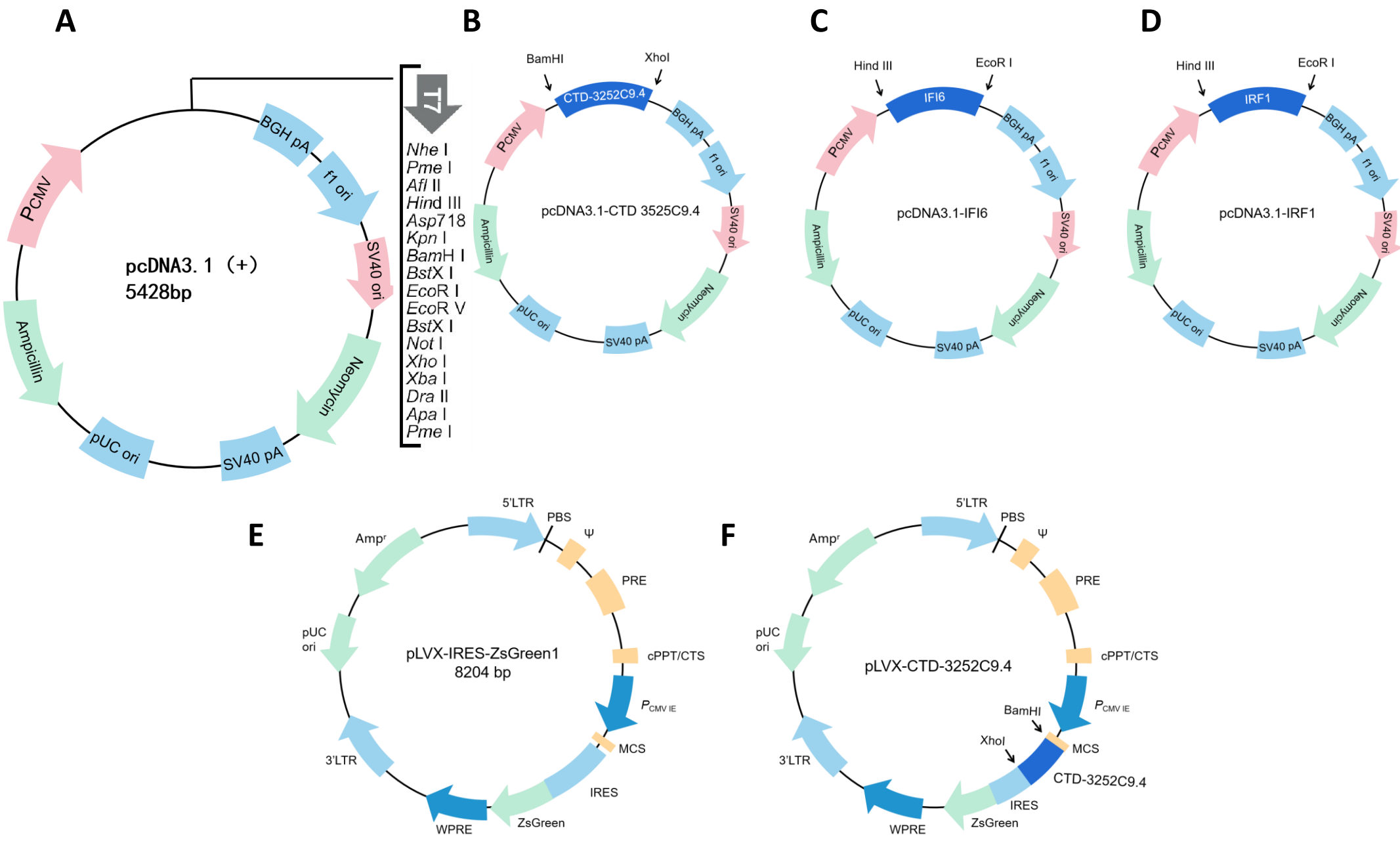

Figure S2

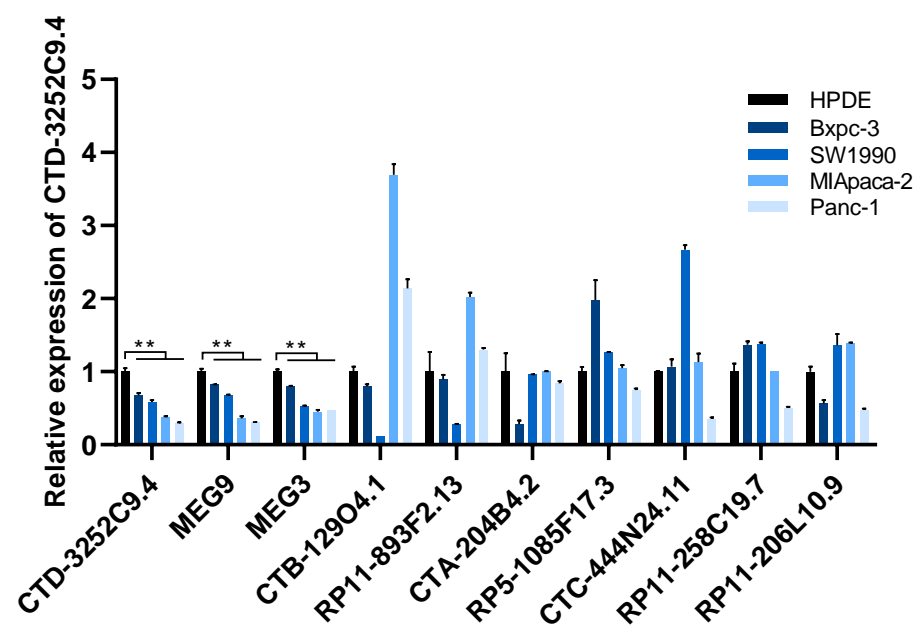

Supplement: Supplementary file 4 — Additional file 5: Fig. S1. Plasmid map. (A-F) Plasmid maps for pcDNA-Ctrl (A), pcDNA-CTD-3252C9.4 (B), pcDNA-IFI6 (C), pcDNA-IRF1 (D), pLVX-Ctrl (E) and pLVX- CTD-3252C9.4 (F). Fig. S2. Relative expression of 10 lncRNAs down-regulated in Panc-1 spheroid cells compared with parental cells were detected in different pancreatic cancer cells. [file 12935_2021_2142_MOESM4_ESM.pdf]
